# The Pyrosequencing-Based Method for *JAK2* Exon 12 Somatic Mutation Detection

**DOI:** 10.3390/diagnostics16121754

**Published:** 2026-06-06

**Authors:** Elena Pozdysheva, Tatiana Subbotina, Yana Voytsekhovskaya, Aleksandra Shalyova, Elena Martynova, Konstantin Mironov, Vasily Akimkin

**Affiliations:** 1 Central Research Institute of Epidemiology Federal Service for Surveillance on Consumer Rights Protection and Human Wellbeing of Russian Federation, 111123 Moscow, Russia; 2 School of Fundamental Biology and Biotechnology, Siberian Federal University, 660041 Krasnoyarsk, Russia; 3 Federal Siberian Research Clinical Center, Federal Medical and Biological Agency, 660037 Krasnoyarsk, Russia; 4 Regional Clinical Hospital, 660022 Krasnoyarsk, Russia

**Keywords:** pyrosequencing, myeloproliferative disease, polycythemia vera, *JAK2* exon 12

## Abstract

**Background**: The detection of *JAK2* exon 12 mutations is important for the differential diagnosis of myeloproliferative neoplasms (MPN) and is included in the World Health Organization’s diagnostic criteria for polycythemia vera (PV). We developed and evaluated a pyrosequencing-based technique to detect somatic mutations in *JAK2* exon 12 and tested the method in a group of PV patients. **Methods**: PCR and pyrosequencing primers were designed for region *JAK2* exon 12; PCR conditions were optimized for subsequent qualitative and quantitative detection by pyrosequencing. Diagnostic specificity and sensitivity were determined using plasmid controls. Genomic DNA from 145 MPN patients was used to validate the method. **Results**: The analytical characteristics of the method were as follows: the limit of blank was 1.2–7.4% and the limit of detection was 3.4–9.9% depending on mutation type. DNA samples from Russian patients with clinical evidence of MPNs were analyzed. In 7 of 145 cases (4.8%) *JAK2* exon 12 mutations were detected. One patient had the mutation I540-E543insdelTCAGAAATG<AAA. The A-homopolimer insertion in this complex mutation type could not be clearly identified by pyrosequencing, and Sanger confirmation was required. For another patient with N542-E543delAATGAA mutation, we observed the mutant allele burden growing from 15 to 55% between 2014 and 2018 and further reduction during treatment to 11%. **Conclusions**: The developed pyrosequencing-based method presents a simple solution to qualitative and quantitative *JAK2* exon 12 mutations detection. However, some complex mutation types may require manual interpretation and Sanger confirmation.

## 1. Introduction

Myeloproliferative neoplasms (MPNs) are a group of clonal hematopoietic stem cell disorders characterized by excessive production of mature myeloid blood cells. At the molecular level, classic MPNs are characterized by the presence of a most common mutation—the *BCR-ABL1* tyrosine kinase fusion gene (Philadelphia chromosome) is formed by the t(9;22) translocation in chronic myeloid leukemia [1]. The patients with *BCR-ABL1*-negative MPNs most often have mutations in the *JAK2* tyrosine kinase gene [2]. These mutations result in the activation of JAK2 kinase leading to activated STAT, AKT, and ERK signaling [3]. The most common mutation for *BCR-ABL1*-negative MPNs is G1849T in exon 14 of gene *JAK2* (*JAK2*V617F) [4]. In addition, mutations found in *JAK2* exon 12 are specific to the single diagnosis of MPN—polycythemia vera (PV) [5,6]. Patients with *JAK2* exon 12-positive PV have a similar clinical course to that of patients with *JAK2*V617F-positive PV, with respect to risk factors such as the development of thrombosis and myelofibrosis [7].

As the finding of *JAK2* mutations confirms the clonal nature of the disease, testing for *JAK2* V617F and exon 12 mutations is now part of the diagnostic protocol for patients with raised hematocrit or hemoglobin and persistent thrombocytosis according to World Health Organization guidelines for the diagnosis of MPNs [8].

The quantitative allele-specific PCR and droplet digital PCR methods are currently recognized as the most appropriate for the detection and monitoring of the mutant allele burden of the single-nucleotide substitution—*JAK2*V617F [9]. At least 37 different *JAK2* exon 12 mutations have been described, residing in a region involving amino acid residues V536-F547 [10,11]. The wide variety of nucleotide substitutions, insertions and deletions makes it difficult to choose a method for identifying *JAK2* exon 12 mutations. In this case, the high-resolution melting (HRM) analysis technique has emerged as superior in sensitivity and convenience in screening of clinical samples for the detection of *JAK2* exon 12 mutations [12,13]. However, the determination of mutation type and mutant allele burden is difficult to apply to this method. High-throughput next-generation sequencing (NGS) is a suitable technology for detecting various types of *JAK2* exon 12 mutations with sufficiently high sensitivity for hematological malignancies [14]. Unfortunately, NGS remains a cost-intensive and technically complex method in the routine practice of many laboratories. Sanger sequencing is widely used to determine and confirm known and novel mutations in *JAK*2 but has a lower analytical sensitivity [15,16].

In this study, we assumed that pyrosequencing may be an optimal technology for sequencing small DNA fragments and identifying various types of mutations (including those not previously described), a more sensitive method than Sanger sequencing, and approaching the sensitivity of NGS.

We configured a pyrosequencing analysis-based method that permitting the detection of the vast majority of *JAK2* exon 12 single-nucleotide substitution, insertions and deletions in region *JAK2* exon 12 involving amino acid residues H531-F547. The diagnostic specificity and sensitivity of some mutation types were determined. We used this method to investigate the genomic DNA from peripheral blood of patients with suspected PV.

## 2. Materials and Methods

### 2.1. Study Samples

Samples for this study were collected at the Krasnoyarsk Regional Clinical Hospital between 2014 and 2025 and were pre-tested for *JAK2*V617F status. *JAK2*V617F preliminary detection was performed using the Real-time-PCR-JAK2-V617F Kit (Syntol, Moscow, Russia). The sensitivity of the kit is 0.2%. Only samples that tested negative for *JAK2*V617F or had a V617F allele burden of less than 1% were further tested for the *JAK2* exon 12 mutation. A total of 189 genomic DNA samples from 145 patients with suspected polycythemia or erythrocytosis of unknown etiology were included in the study. Among the 145 patients who participated in the study, 5 cases had *JAK2*V617F < 1% (4.1%). In addition, all samples were tested using methods for screening *JAK2* exon 12 mutations based on heteroduplex and HRM analysis, as described in [17].

All patients had elevated hemoglobin values (for women > 160 g/L, for men > 165 g/L) and/or elevated hematocrit values (>48% in women, >49% in men). Among the patients, there were 30 women (mean age 51.8 ± 14.7 years) and 115 men (mean age 46.7 ± 13.7 years).

All patients had given informed consent.

### 2.2. Ethics Approval

The study was approved by the local biomedical Ethics Committee of the Federal Siberian Research Clinical Center, Federal Medical and Biological Agency of Krasnoyarsk (protocol number 1, 2 September 2014) and was conducted in accordance with the Declaration of Helsinki. All participants signed informed consent statements prior to sample collection.

### 2.3. Cloned Template Design

Cloned template controls with seven different mutation types were used to develop the method (Table 1). Patient DNA with a confirmed mutation or wild-type DNA was used as a template for cloning.

Five plasmid controls were cloned using PCR amplicons. Patient DNA with confirmed mutations or wild-type DNA was used as a template. Each PCR product was ligated into pGem-T vector (Promega, Madison, WI, USA) and transformed into competent *Escherichia coli* cells (strain DH10B). The produced clones were screened for PCR and appropriate plasmids were isolated using the GeneJET Plasmid Miniprep Kit (Thermo Fisher Scientific, Waltham, MA, USA). DNA of the positive colonies was sequenced using M13 primers with BigDye Terminator V1.1 chemistry according to supplier’s recommendations on an ABI 3500xL genetic analyzer (Thermo Fisher Scientific, Waltham, MA, USA).

Plasmids p.H538_K539delinsQL and p.K539L were produced by the site-directed mutagenesis method using the QuikChange II Site-Directed Mutagenesis Kit (Agilent Technologies, Santa Clara, CA, USA). The plasmid sequences were confirmed by Sanger sequencing.

All cloned controls were measured using real-time PCR, employing primers specific to the pGem-T vector sequence, calibrator reagents from the Central Research Institute of Epidemiology and RotorGene instrumentation (Qiagen, Hilden, Germany).

### 2.4. DNA Extraction

The isolation of DNA from 250 μL whole-blood samples was performed using the DNA-Sorb-B Nucleic Acid Extraction Kit (AmpliSens, Moscow, Russia) according to the manufacturer’s protocol. Quality and concentration were assessed with a NanoDrop 1000 spectrophotometer (Thermo Fisher Scientific, Waltham, MA, USA). Subsequent storage of the isolated DNA was conducted at −20 °C.

### 2.5. PCR and Pyrosequencing

For the PCR reaction and pyrosequencing, primers were designed with the PyroMark Assay Design Software v2.0 (Qiagen, Hilden, Germany). The PCR reaction was performed in a 25 μL tube containing 10 μL of DNA (~3000 copies/reaction), 9.5 μL of 2.5xPCR buffer blue (AmpliSens, Moscow, Russia), 0.5 μL of TaqF polymerase (AmpliSens, Moscow, Russia), 0.88mM dNTP, 7 pmol forward primer (5′-CTCACCAACATTACAGAGGCCTACT-3′) and 7 pmol reverse primer (5′-biotin-GTCACATGAATGTAAATCAAGAAAACAGATG-3′). The PCR reaction was performed with an annealing temperature of 60 °C for 45 cycles. A *JAK2* exon 12 mutation-negative plasmid control was included on each run. The PCR product (5 μL) was prepared for subsequent analysis using the Pyro-Prep Reagent Kit (AmpliSens, Moscow, Russia). The PCR products were sequenced three times using two sequencing primers and three nucleotide dispensation orders (Supplementary Materials Table S1).

All dispensation orders were generated according to the reference sequence of *JAK2* exon 12 and potential mutations within H531-F547 coding region of the Catalogue of Somatic Mutations in Cancer (COSMIC) database [18]. The order of addition of nucleotides to the reaction mixture was determined by adding a conservative nucleotide sequentially to a given position, followed by the addition of a mutant nucleotide if its presence was expected (Supplementary Materials Table S1).

The sequencing reaction was performed on a PyroMark Q24 using the Pyro Gold Reagent Kit (Qiagen, Hilden, Germany), according to the manufacturer’s instructions. The PyroMark Q24 v2.0.8 software processed the assay designs and resulting pyrograms. Sequencing primer sequences and the analysis sequences are given in Supplementary Materials Table S1.

### 2.6. Analytical Characteristics of the Method

The analytical characteristics: the limit of blank—LOB (the highest signal expected to be found when a blank sample containing no analyte is tested):

LOB = M + 1.645 × *σ*,

where M and *σ* are the mean and standard deviations of the signal values in a batch of wild samples, respectively, and the limit of detection (LOD) (the lowest analyte concentration likely to be reliably distinguished from the LOB value):

LOD = LOB + 1.645 × *σ*,

where *σ* is the standard deviation of the signal values in a batch of samples with a mutation; the analytical characteristics were measured as described in our earlier studies [19,20].

The analytical characteristics of the developed methods were determined using dilutions of plasmid DNA samples containing the *JAK2* exon 12 region cloned into the pGem-T vector, wild or having one of the mutations (Table 1). Wild-type or mutant alleles were analyzed by mixtures containing 0, 1, 2, 3, 4, 5, 6, 7, 10, 20 and 30% of the mutant allele. All mixtures of the mutant allele were tested at least six times. A cloned wild-type *JAK2* exon 12 was tested at least 24 times.

### 2.7. Statistical Analysis

Microsoft Excel was used for all data processing steps, tables organization, calculation of the main analytical indicators (LOB, LOD), and figure plotting. Linear regression analysis and data confirmation were performed with XLMiner Analysis ToolPak (version 2.0.0.0).

## 3. Results

### 3.1. Design of Pyrosequencing Assay for JAK2 Exon 12 Somatic Mutation Detection

The COSMIC database was searched to identify published *JAK2* exon 12 mutations within the H531-F547 coding region. The 46 most common somatic mutations were selected. Assays were developed for detection of selected mutations using two primers: additional nucleotides were added to the reference sequence of dispensation orders to account for all 46 possible mutation variants.

The first sequence including the H531-I540 coding region was analyzed using primer 5′-CTCACCAACATTACAGAGGCCTACT-3′ and nucleotide dispensation order GCTATCGATGTACAATACGTGCTTCACAAT. The next sequence including V536-F547 coding region was analyzed using primer 5′-GCCTACTCATATGAACCAAATGG-3′ in two sequence reaction with the following nucleotide dispensation orders: CTGTTCACAATCAGAACTGAGATTGATATTGTAGT and CTGATTCAGTCTGAATACAGAATGAGATGTCGATACGTCTGTAGT (Supplementary Materials Table S1).

The percentage of mutant versus wild-type alleles was analyzed using allele quantification (AQ) PyroMark Q24 v2.0.8 software: to do this, nucleotide sequences with suitable variable positions were entered one by one into the “Sequence to Analyze” software (version 2.0.8) line and were analyzed manually. Detailed information on the dispensation orders and sequences is provided in Supplementary Materials Table S1.

### 3.2. Analytical Characteristics of JAK2 Exon 12 Mutant Allele Burden Determination

To determine the LOB and LOD of the developed method, we prepared serial dilutions of *JAK2* exon 12 mutation plasmid controls in wild-type plasmid control and then subsequently determined mutant allele burdens using the developed protocol. The LOB level of wild-type control in the analysis sequences ranged from 1.2 to 7.4%. For different mutation types, the LOD level ranged from 3.4 to 10.0%. The analytical characteristics are shown in Table 2.

**Figure 1:**
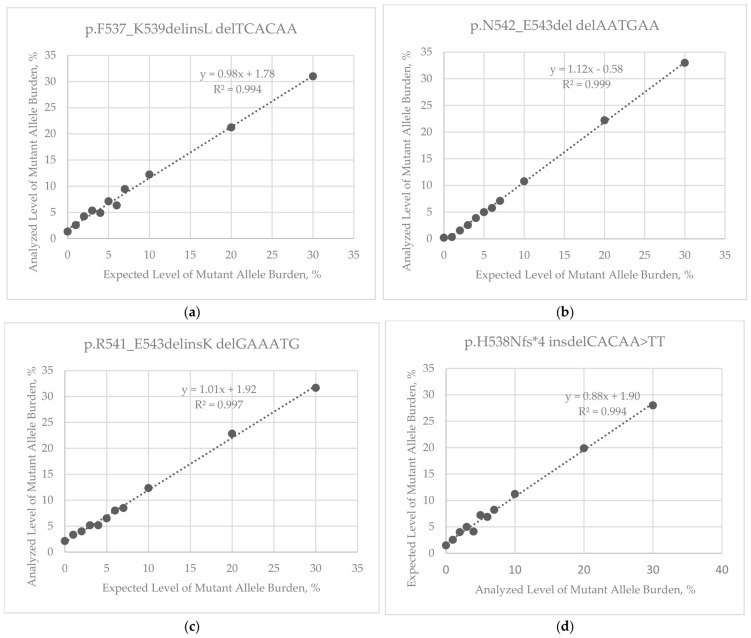

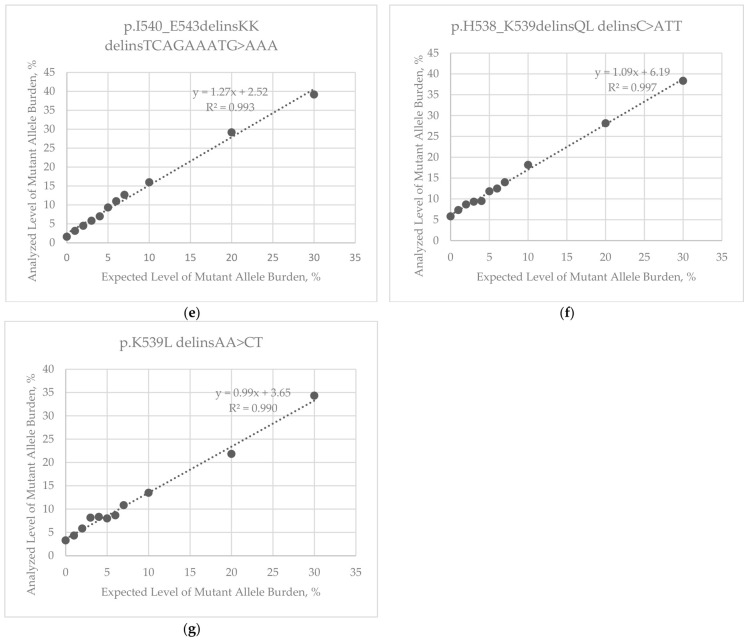
Linear relationship between percentages of expected and analyzed level of *JAK2* exon 12 mutant allele burden by pyrosequencing: *x*-axis—the mutant allele burden percentage in the prepared control samples; *y*-axis—detected mutant allele burden percentage. R^2^—coefficient of determination. (**a**) linear relationship between expected (x-axis) and analyzed level (y-axis) of p.F537_K539delinsL delTCACAA mutant allele burden; (**b**) linear relationship between expected (x-axis) and analyzed level (y-axis) of p.N542_E543del delAATGAA mutant allele burden; (**c**) linear relationship between expected (x-axis) and analyzed level (y-axis) of p.R541_E543delinsK delGAAATG mutant allele burden; (**d**) linear relationship between expected (x-axis) and analyzed (y-axis) level of p.H538Nfs*4 insdelCACAA>TT mutant allele burden; (**e**) linear relationship between expected (x-axis) and analyzed (y-axis) level of p.I540_E543delinsKK delinsTCAGAAATG>AAA mutant allele burden; (**f**) linear relationship between expected (x-axis) and analyzed (y-axis) level of p.H538_K539delinsQL delinsC>ATT mutant allele burden; (**g**) linear relationship between expected (x-axis) and analyzed (y-axis) level of p.K539L delinsAA>CT mutant allele burden.

The dependences of the actual percentages of mutant allele burdens in the analyzed control samples on the observed values were described by linear regressions (Figure 1). The correlation coefficients ranged from 0.990 to 0.999.

### 3.3. Identification of JAK2 Exon 12 Mutations and Quantitative Determination of Mutant Allele Burden in the Patients

In the studied cohort, seven patients (4.8% of all cases, n = 145) were found *JAK2* exon 12-positive using pyrosequencing. Three patients with the N542-E543del mutation were identified; four patients with various mutations including p.F537_K539delinsL, p.H538Nfs*4, p.R541_E543delinsK and p.I540_E543delinsKK were identified (Supplementary Materials Figure S1). Amplified *JAK2* fragments for these patients were cloned and mutations were confirmed by Sanger sequencing.

According to the manufacturer’s guidelines, analysis of homopolymers or combined insertions/deletions is a limitation of the pyrosequencing method. In this study, the Sanger confirmation step was necessary to confirm the correct interpretation of the results obtained pyrosequencing. We correctly interpreted all identified mutations except for one case: I540-E543insdelTCAGAAATG<AAA. Quantitative analysis of the change in the peak height of A at position 9 (Supplementary Materials Figure S1e) was not possible because the mutant variant contained -(A)_9_-homopolymer.

**Figure 2:**
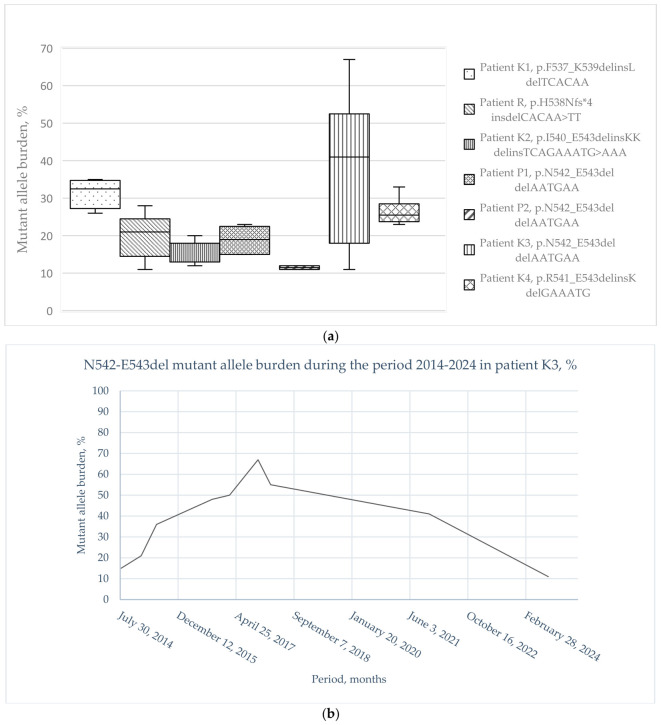
The distribution of mutant allele burden in the *JAK2* exon 12-positive patients: (**a**) observation of the allele burden in various DNA samples from seven patients over the period from 2014 to 2025 measured by pyrosequencing; (**b**) dynamics of the mutant allele burden alteration of patient K3 with N542-E543del mutation.

In addition, the patient with mutation I540_E543delinsKK also had the *JAK2*V617F mutation: the V617F allele burden was 0.37%, TCGAAAATG<AAA—14.9%. The mutant allele burdens of other mutations ranged from 11 to 67%. To investigate the development of the mutated clones, variations in the mutant allele burdens were measured for all *JAK2* exon 12 positive patients during the study. The median follow-up time for patients with *JAK2* exon 12 mutations was 5.1 years: the shortest observation period was 6 months for patient P2; the longest observation period was 10 years for patient K3. Histogram plots showing the scatter of the mutant allele burden for seven patients are shown in Figure 2a. We observed a significant allele burden change in patient K3 with N542-E543del mutation between 2014 and 2024. At the beginning of the patient’s observation, the allele burden grew from 15% to 67%, with a loss of heterozygosity. When hydroxyurea therapy was started in 2018, it began to decrease, and at the latest measurement in 2024, it was 11% (Figure 2b).

The results obtained are in agreement with the results from additional screenings using heteroduplex and HRM analysis, which were performed on all 145 patients [17]. The sensitivity threshold of the heteroduplex analysis was 3.13–6.25% allele burdens, the sensitivity threshold of the HRM assay was 6.25–12.5%, depending on the mutation. The sensitivity of all three approaches—heteroduplex analysis, HRM analysis, and pyrosequencing—proved to be comparable, yet it was not possible to identify the types of detected mutations and the mutant allele burden using heteroduplex or HRM analysis methods. The results of comparing all methods are presented in Table 3.

### 3.4. Clinical Characteristics of Patients with JAK2 Exon 12 Mutations

Clinical and laboratory data were collected from the time of initial diagnosis to the present. Some clinical and laboratory characteristics of seven patients with *JAK2* exon 12 mutations are reported in Table 4.

The subjects were 115 men and 30 women (the male-to-female ratio was 3.8). The age of disease onset for the subjects ranged from 28 to 72 years, with a median age of 45.1 ± 17.8 years for patients with exon 12 mutations. Three out of these patients became ill before the age of 30. The duration of the disease ranged from two to twenty years. All 7 patients showed an increase in red blood cell count, accompanied by increased hemoglobin concentration and hematocrit levels in the peripheral blood. Some of them had moderate increase number of leukocytes and platelets in the disease dynamics. Patients K1, K2, K3, P1 and R had splenomegaly (71%) in the anamnesis. Patients K1, K2, K4 and P2 in the anamnesis had thrombotic events (57%—4 out of 7). Patients K1, K3, R and P2 are receiving hydroxyurea therapy. Patient K4 refused cytoreductive therapy. Patient P1 received interferon, then hydroxyurea, and is currently taking ruxolitinib. The subjects were 115 men and 30 women (the male-to-female ratio was 3.8).

## 4. Discussion

In this study, we use the pyrosequencing technique to detect mutations region of *JAK2* exon 12 involving the H531-F547 coding region (~50bp). We previously demonstrated the possibility of using this technology for analysis of the *JAK2*V617F mutation [18] and *BRAF* gene [19].

It was found that the pyrosequencing LOD of *JAK2* exon 12 mutant allele burden ranged from 3.42 to 9.97% in the analyzed samples (mean = 6.6%). This is consistent with previously published data showing that pyrosequencing has a sensitivity of 5% for detecting mutations in *BRAF* and *KRAS* genes, for example, [21,22]. Herewith, as our research showed, the LOD is highly dependent on the mutation type. Mutations that are simpler in terms of nucleotide sequence, such as N542-E543del and p.R541_E543insK, show the highest sensitivity of LOD. The LOD of p.I540_E543delinsKK delinsTCAGAAATG>AAA also turned out to be sensitive, but difficulties arose when analyzing mutations of this type. Specifically, the allele quantification analysis software was unable to perform simultaneous analysis of delTCAGAAATG and insAAA due to the proximity of the variable position TCAGAAATG to the homopolymer A. The pyrograms were flagged as abnormal, but the pattern could not be definitively interpreted with regard to the specific mutation subtype. The solution was a quantitative analysis of only delTCAGAAATG with the determination of % mutant allele burden, but not quantitative analysis of the change in peak height of the homopolymer. It was not possible to determine the exact number of “A” nucleotides incorporated, and therefore this was not carried out (see Supplementary Materials Figure S1e). In such a case, determining the mutation type for pyrosequencing might have been somewhat subjective. Insertion AAA within the -(A)_9_-homopolymer has been accurately identified through Sanger sequencing and preliminary sample cloning. Our findings indicate that pyrosequencing is not always a unique method. Detecting complex mutations may require the use of additional techniques and protocols.

Various sequencing methods have become an indispensable diagnostic tool for identifying somatic mutations. However, in cancer diagnostics, detecting low-level somatic variants is particularly challenging due to tumor heterogeneity and contamination from normal cells. For research purposes, when choosing a clinical testing platform, factors to consider include sensitivity, specificity, reproducibility, turnaround time, ease of interpretation, and cost.

It is worth noting that using the classic Sanger “gold sequencing standard” to detect *JAK2* exon 12 somatic mutations seems difficult. The sensitivity of Sanger sequencing has been described in the literature as 20% mutated alleles in a background of wild-type alleles [23], although later studies have demonstrated a decrease in the threshold to up to 10% [22]. Nevertheless, interpretations with a threshold of 10% or lower often lead to both false positive and false negative mutation calls. This highlights the need for careful verification of all sequencing data using the Sanger method for samples with a mutant allele burden ≤ 10%. Further reduction in the threshold is only possible using the wild-type blocking technologies [24].

Published data using targeted NGS to detect *JAK2* exon 12 mutations demonstrate sensitivity similar to pyrosequencing. According to published data, NGS sensitivity in detecting *JAK2* exon 12 mutations is 3–6% [25,26]. In addition, NGS may be preferable to pyrosequencing for analyzing longer DNA fragments. However, the application of NGS presents challenges: sequencing and analysis errors remain major barriers to interpretation of results. So, for example, the results of comparing different certified NGS providers demonstrate that analytical sensitivity differs by up to 13.9-fold, and false positive error rates vary up to 615-fold, depending on provider and pipeline. For identical raw data, pipelines differ by up to 36.3-fold in false positive error rates [27].

In our opinion, pyrosequencing has proven to be a convenient method for the detection of *JAK2* exon 12 mutations. To our knowledge, this is the only study to have used pyrosequencing to determine the LOB and LOD levels of *JAK2* exon 12 mutations. In our study, pyrosequencing seems to be somewhere in between Sanger sequencing and NGS in terms of sensitivity and specificity. Although pyrosequencing may require confirmatory testing in some cases, we found the overall interpretation of pyrosequencing data to be straightforward.

In addition, our study demonstrated that the results from pyrosequencing were 100% consistent with the data from screening clinical samples using heteroduplex and HRM analysis [17]. Our experience shows that the developed method can be used as an independent method or in combination with other screening methods as a supplementary and confirmatory measure.

In this study, we tested a cohort of patients with suspected polycythemia using the developed method and identified 4.8% cases of *JAK2* exon 12 mutations. This is consistent with data from Western countries, where approximately 2–5% of patients with PV have *JAK2* exon 12 mutations [5,28,29].

In our study, we observed an earlier age of disease onset (median age −45 years) in patients with *JAK2* exon 12 mutations compared to epidemiological data on the median age of PV [30]. Three out of these patients became ill before age 30. However, an earlier onset of disease was reported in patients with *JAK2* exon 12 mutations [31,32]. It can be assumed that *JAK2* exon 12 mutations shift the onset of PV to an earlier age.

As is known, erythrocytosis is a common feature of patients with PV with *JAK2* exon 12 mutations. In this study, all patients also had erythrocytosis. Splenomegaly was observed in 71% of patients with *JAK2* exon 12 mutations; our findings are consistent with a previous report from a large cohort of patients in Taiwan [32]. Thrombosis was registered in the history and/or at the time of diagnosis in four patients (57%). These figures are higher than those in other cohorts studied. There are data on different frequencies of thrombotic events in PV in different populations: from 12 to 30% in Asian countries and from 5 to 42% in Western countries [31,33,34,35,36].

At the time of writing, none of the patients with *JAK2* exon 12 mutations had developed acute myeloid leukemia, which is consistent with the published data [32] and contradicts the results of previous European studies [36,37].

We observed significant changes in *JAK2* exon 12 mutant allele burden and the loss of heterozygosity in only one patient. This patient did not show any clinical differences compared to other patients with an allele burden < 50%. However, when the allele burden exceeded 50%, the patient’s condition worsened, and hydroxyurea therapy was started. During treatment, the allele burden decreased, demonstrating the importance of monitoring the mutant allele burden. Our results showed that pyrosequencing is sufficiently accurate and reproducible to measure allele burden and undoubtedly improves the monitoring of therapy’s impact on patients.

The remaining six patients with mutations were also prescribed optimal therapy in accordance with current guidelines [38]. However, it was not effective for all; for example, patient P1 had to undergo several treatment changes due to showing no improvement. At the time of writing, none of the six patients showed any changes in allele load during therapy.

Our results demonstrate the potential of using the developed pyrosequencing-based method to analyze *JAK2* exon 12 mutations. This method may contribute to the need for further study of the prognostic significance of allele burden in *JAK2* exon 12-positive patients.

The results obtained for *JAK2* exon 12 mutations confirm the feasibility of using pyrosequencing to detect various somatic mutations. This protocol can be used to detect other somatic mutations. However, as our results show, the pyrosequencing method is not a reliable way to detect complex types of mutations, such as insertions/deletions and homopolymers. In some cases, it may require the use of additional confirmatory methods, particularly when identifying new, previously unknown variants.

## Figures and Tables

**Table 1:** Unique ID, the mutation syntax at the amino acid and nucleotide sequence of *JAK2* exon 12.

Genomic Mutation ID	Predicted Amino Acid Change	cDNA Mutation
COSV6760884	p.H538_K539delinsQL	c.1614_1616delinsC>ATT
COSV67589923	p.K539L	c.1615_1616delinsAA>CT
COSV67579858	p.F537_K539delinsL	c.1611_1616delTCACAA
COSV67595506	p.H538Nfs*4	c.1612_1616delinsCACAA>TT
COSV67586963	p.R541_E543delinsK	c.1622_1627delGAAATG
COSV67575778	p.N542_E543del	c.1624_1629delAATGAA
COSV67625452	p.I540_E543delinsKK	c.1619_1627delinsTCAGAAATG>AAA

**Table 2:** Measured % mutant allele burden in wild-type control, LOB and LOD of the developed method.

Mutation Type	Mean Background Value ± *σ*	Limit of Blank (LOB), %	Limit of Detection (LOD), %
p.H538_K539delinsQL	5.83 ± 0.96	7.42	9.89
p.K539L	3.29 ± 2.20	6.90	9.97
p.F537_K539delinsL	1.37 ± 1.62	4.03	8.31
p.H538Nfs*4	1.50 ± 1.40	3.80	5.72
p.R541_E543delinsK	2.13 ± 0.62	3.16	4.39
p.N542_E543del	0.19 ± 0.60	1.18	4.41
p.I540_E543delinsKK	1.62 ± 0.55	2.52	3.42

**Table 3:** Comparison of the heteroduplex analysis, HRM analysis, and pyrosequencing for identification of *JAK2* exon 12 mutations in the analyzed patient cohort.

Parameter	Heteroduplex Analysis	High-Resolution Melt (HRM) Analysis	Pyrosequencing
Method characteristics:			
Number of steps	2 (PCR, electrophoresis)	1 (PCR)	2 (PCR, pyrosequencing)
Identification of mutation type	No	No	Yes ^1^
Quantitative determination of allele burden	No	No	Yes ^1^
Number of patients:			
Negative cases	138	138	138
Positive cases	7	7	7
Sensitivity of method, %:			
p.F537_K539delinsL	3.13	12.5	8.31
p.H538Nfs*4	3.13	12.5	5.72
p.R541_E543delinsK	6.25	12.5	4.39
p.N542_E543del	3.13	12.5	4.41
p.I540_E543delinsKK	6.25	6.25	3.42

^1^ Complex cases may require additional confirmation.

**Table 4:** Clinical characteristics of *JAK2* exon 12-positive patients.

Patient	Sex/Disease	*JAK2* 12 Exon Mutation	Age at Onset of PV, (Years)	PV Duration (Months)/Disease Outcome	Cytoreductive Treatment	*JAK2* V617F	Hb Max, g/dL	HCT Max, %	RBC Max, 10^12^/L	WBC Max, 10^9^/L	PLT Max, 10^9^/L	Thrombotic Events
K1	M/PV	p.F537_K539delinsL delTCACAA	29	216/ Alive	HU	Neg	17.8	56.0	5.6	13.5	436	Yes
R	F/PV	p.H538Nfs*4 insdelCACAA>TT	28	180/ Alive	HU	Neg	19.8	48.8	5.9	7.7	302	No
K2	M/PV	p.I540_E543delinsKK del ins TCAGAAATG>AAA	48	84/ Lost to follow-up	No	<1%	24.2	71.0	8.9	9.2	198	Yes
P1	M/PV	p.N542_E543del delAATGAA	27	240/ Alive	interferon, HU, ruxolitinib	Neg	16.4	60.9	8.9	6.1	560	No
P2	M/PV	p.N542_E543 delAATGAA	51	26/ Alive	HU	Neg	18.3	65.2	9.11	7.9	281	Yes
K3	M/PV	p.N542_E543 delAATGAA	61	150/ Alive	HU	Neg	20.4	71.5	9.0	11.4	500	No
K4	F/PV	p.R541_E543delinsK delGAAATG	72	108/ Lost to follow-up	No	Neg	17.0	51.0	8.6	10.0	486	Yes

## Data Availability

The original contributions presented in this study are included in the article and Supplementary Materials. Further inquiries can be directed to the corresponding author.

## Supplementary Materials

The following supporting information can be downloaded at https://www.mdpi.com/article/10.3390/diagnostics16121754/s1, Table S1: Sequencing primer sequences, nucleotide dispensation orders and the analysis sequences for mutations; Figure S1: Identification of *JAK2* exon 12 mutations by pyrosequencing.

## Abbreviations

The following abbreviations are used in this manuscript:

MPN	Myeloproliferative neoplasm
PV	Polycythemia vera
BCR-ABL1	Tyrosine kinase fusion gene (Philadelphia chromosome)
STAT	Signal Transducer and Activator of Transcription signaling pathway
AKT	serine/threonine kinase signaling pathway
ERK	Extracellular signal-regulated kinase signaling pathway
HRM	High-resolution melting analysis technique
LOB	Limit of blank
LOD	Limit of detection
HU	Hydroxyurea
Hb	Hemoglobin count
HCT	Hematocrit count
RBC	Red blood cells count
WBC	White blood cell count
PLT	Platelet count
