# Supplementary material for: The Pyrosequencing-Based Method for JAK2 Exon 12 Somatic Mutation Detection
Source: Diagnostics (Basel). 2026 Jun 6;16(12):1754. doi: 10.3390/diagnostics16121754 (PMC13298268; doi:10.3390/diagnostics16121754)
Supplement: Supplementary file 1 [file diagnostics-16-01754-s001.zip › diagnostics-4319261-supplementary.pdf]

Supplementary Table S1. Sequencing primer sequences, nucleotide dispensation orders and the analysis sequences for *JAK2* exon 12 mutations.

Sequencing primer, 5′→3′	Dispensation Order*	Sequence to Analyze**	Targeted Mutation	Genomic Mutation ID
CTCACCAACATTACA GAGGCCTACT	GC**T**AT**CG**ATG**T**ACAAT**AC**GTG**C** TTCACAAT	C/TATATGAACCAAATGGTGTTTCACAAAATCAG	c.1591C>T	COSV107501394
		CT/ATATGAACCAAATGGTGTTTCACAAAATCAG	c.1592A>T	COSV101077868
		CAT/CATGAACCAAATGGTGTTTCACAAAATCAG	c.1593T>C	COSV67616199
		CAT[A]TGAACCAAATGGTGTTTCACAAAATCAG	c.1594_1595del	COSV106113122
		CATATGG/AACCAAATGGTGTTTCACAAAATCAG	c.1597A>G	COSV67573327
		CATATGT/AACCAAATGGTGTTTCACAAAATCAG	c.1597A>T	COSV67681304
		CATATGAACCAAATNGTGTTTCACAAAATCAG	c.1605G>A	COSV67614088
			c.1605G>C	COSV67602511
			c.1605G>T	COSV67626091
GCCTACTCATATGAA CCAAATGG	CTGTTCACAATCAGAA**C**TGAGA TTGATATTGTAGT	TGTT[TCACAA]AATCAGAAATGAAGATTTGATATTTGTAAGTC ATT	c.1611_1616del	COSV67579858
		TGTTT[CACAAA]ATCAGAAATGAAGATTTGATATTTGTAAGTC ATT	c.1612_1617del	COSV67667793
		TGTTT[T][CACAA]AATCAGAAATGAAGATTTGATATTTGTAAGT CATT	c.1612_1616del	COSV67595506
		TGTTT[CAC]AAAATCAGAAATGAAGATTTGATATTTGTAAGTC ATT	c.1612_1614del	COSV67639238
		TGTTTCA[CAA]AATCAGAAATGAAGATTTGATATTTGTAAGTC ATT	c.1614_1616del	COSV67685250
		TGTTTCACAAAA[TCAGAAATG]AAGATTTGATATTTGTAAGTC ATT	c.1619_1627delins	COSV67625452
		TGTTTCACAAAAT[C]AGAAATGAAGATTTGATATTTGTAAGTC ATT	c.1620_1621del	COSV67652869
		TGTTTCACAAAAT[CAGAAATGAA]GATTTGATATTTGTAAGTC ATT	c.1620_1629del	COSV67575639
		TGTTTCACAAAATCA[GAAATG]AAGATTTGATATTTGTAAGTC ATT	c.1622_1627del	COSV67586963
		TGTTTCACAAAATCAGA[AATGAA]GATTTGATATTTGTAAGTC ATT	c.1624_1629del	COSV67575778
		TGTTTCACAAAATCAGAAA[C][TGAAGAT]TTGATATTTGTAAG TCATT	c.1626_1632delins	COSV67686535
		TGTTTCACAAAATCAGAAA[TGAA]GATTTGATATTTGTAAGTC ATT	c.1626_1629del	COSV67601476

		TGTTTCACAAAATCAGAAAT[GAAGAT]TTGATATTTGTAAGTC ATT	c.1627_1632del	COSV67569287
	CTGATTCAGTCTGAATACAGAA TGAGATGTCGATACGTCTGTAG T	TGA/TTTCACAAAATCAGAAATGAAGATTTGATATTTGTAAGT	c.1609T>A	COSV67645975
		TGTTTT/CACAAAATCAGAAATGAAGATTTGATATTTGTAAGT	c.1612C>T	COSV67602516
		TGTTTCAC/AAAAATCAGAAATGAAGATTTGATATTTGTAAGT	c.1614C>A	COSV67575797
		TGTTTCAC/GAAAATCAGAAATGAAGATTTGATATTTGTAAGT	c.1614C>G	COSV108231723
		TGTTTCA[GTT]CAAAATCAGAAATGAAGATTTGATATTTGTAA GT	c.1614_1616delins	COSV67626740
		TGTTTCA[TTT]CAAAATCAGAAATGAAGATTTGATATTTGTAAG T	c.1614_1616delins TTT	COSV104701491
		TGTTTCA[ATT]CAAAATCAGAAATGAAGATTTGATATTTGTAA GT	c.1614_1616delins ATT	COSV67608841
		TGTTTCAC[TT][A]AAATCAGAAATGAAGATTTGATATTTGTAAG T	c.1615_1616ins	COSV67575806
		TGTTTCACA/TAAATCAGAAATGAAGATTTGATATTTGTAAGT	c.1615A>T	COSV67615657
		TGTTTCAC[CT][A]AAAATCAGAAATGAAGATTTGATATTTGTA AGT	c.1615_1616delins CT	COSV67589923
		TGTTTCACA/GAAATCAGAAATGAAGATTTGATATTTGTAAGT	c.1615A>G	COSV67595951
		TGTTTCACA[A]AA[T]TCAGAAATGAAGATTTGATATTTGTAAGT	c.1616A>T	COSV67645980
		TGTTTCACAAAA/TTCAGAAATGAAGATTTGATATTTGTAAGT	c.1619T>A	COSV67614545
		TGTTTCACAAAAT/CCAGAAATGAAGATTTGATATTTGTAAGT	c.1619T>C	COSV67649475
		TGTTTCACAAAATT/CAGAAATGAAGATTTGATATTTGTAAGT	c.1620C>T	COSV67665774
		TGTTTCACAAAATCAG/AAAATGAAGATTTGATATTTGTAAGT	c.1622G>A	COSV107501320
		TGTTTCACAAAATCAGAAATG/AAAGATTTGATATTTGTAAGT	c.1627G>A	COSV107501492
		TGTTTCACAAAATCAGAAATGAAGG/ATTTGATATTTGTAAGT	c.1631A>G	COSV67626850
		TGTTTCACAAAATCAGAAATGAAGATT/GTGATATTTGTAAGT	c.1633T>G	COSV67629634
		TGTTTCACAAAATCAGAAATGAAGATT[T][C]GATATTTGTAAG T	c.1634T>C	COSV67665784
		TGTTTCACAAAATCAGAAATGAAGATTTC/GATATTTGTAAGT	c.1635G>C	COSV67632570
		TGTTTCACAAAATCAGAAATGAAGATTTGATAC/TTTGTAAGT	c.1639T>C	COSV67590082
		TGTTTCACAAAATCAGAAATGAAGATTTGATAG/TTTGTAAGT	c.1639T>G	COSV67584941
		TGTTTCACAAAATCAGAAATGAAGATTTGATATT/CTGTAAGT	c.1641T>C	COSV67625116

* The dispensation orders were generated according to the reference sequence of JAK2 exon 12 within H531-F547 coding region from the (COSMIC) database, and in accordance with the manufacturer's guidelines. Additional nucleotides have been added in Dispensation Order to accommodate various mutation variants (marked in red). It is necessary to sequence the PCR product once for further analysis using the primer CTCACCAACATTACAGAGGCCTACT, and using assay with dispensation order

GCTATCGATGTACAATACGTGCTTCACAAT (the section Sequence to Analyze in AQ Assay (PyroMark Q24 v2.0.8 software) should be left blank). Then, to sequence the PCR product twice using the primer GCCTACTCATATGAACCAAATGG, and using two assays with different dispensation orders: CTGTTCACAATCAGAACTGAGATTGATATTGTAGT and CTGATTCAGTCTGAATACAGAATGAGATGTCGATACGTCTGTAGT.

** After sequencing is complete, the pyrogram analysis must be performed. To do this, sequences with different mutations are inserted in a specific dispensation order (the "Sequence to Analyze" section in AQ Assay (PyroMark Q24 v2.0.8 software) must be filled in). The PyroMark Q24 v2.0.8 software automatically performs qualitative and quantitative analysis of the variable nucleotide region.

Supplementary Figure S1. Identification of *JAK2* exon 12 mutations by pyrosequencing.

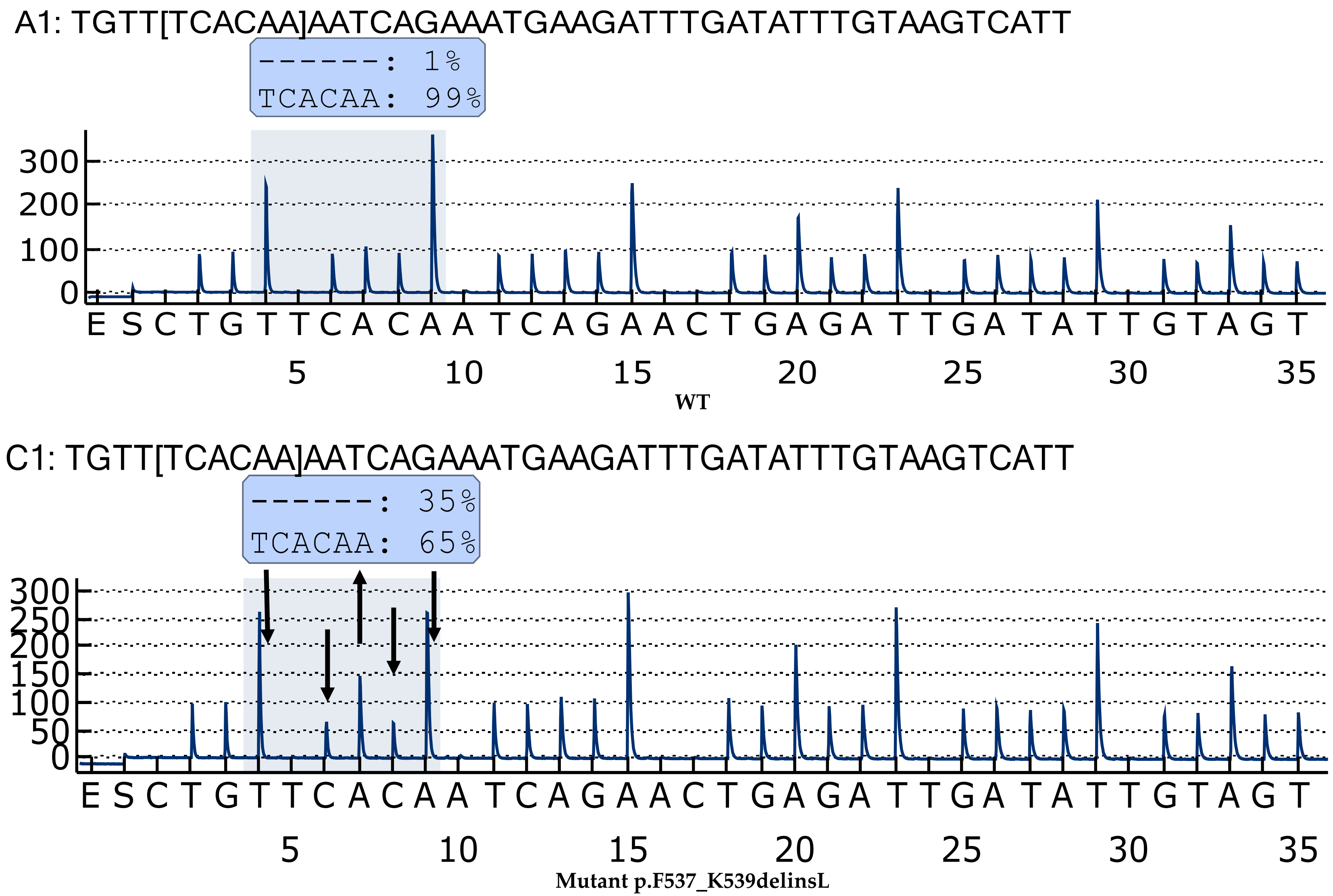

(a)

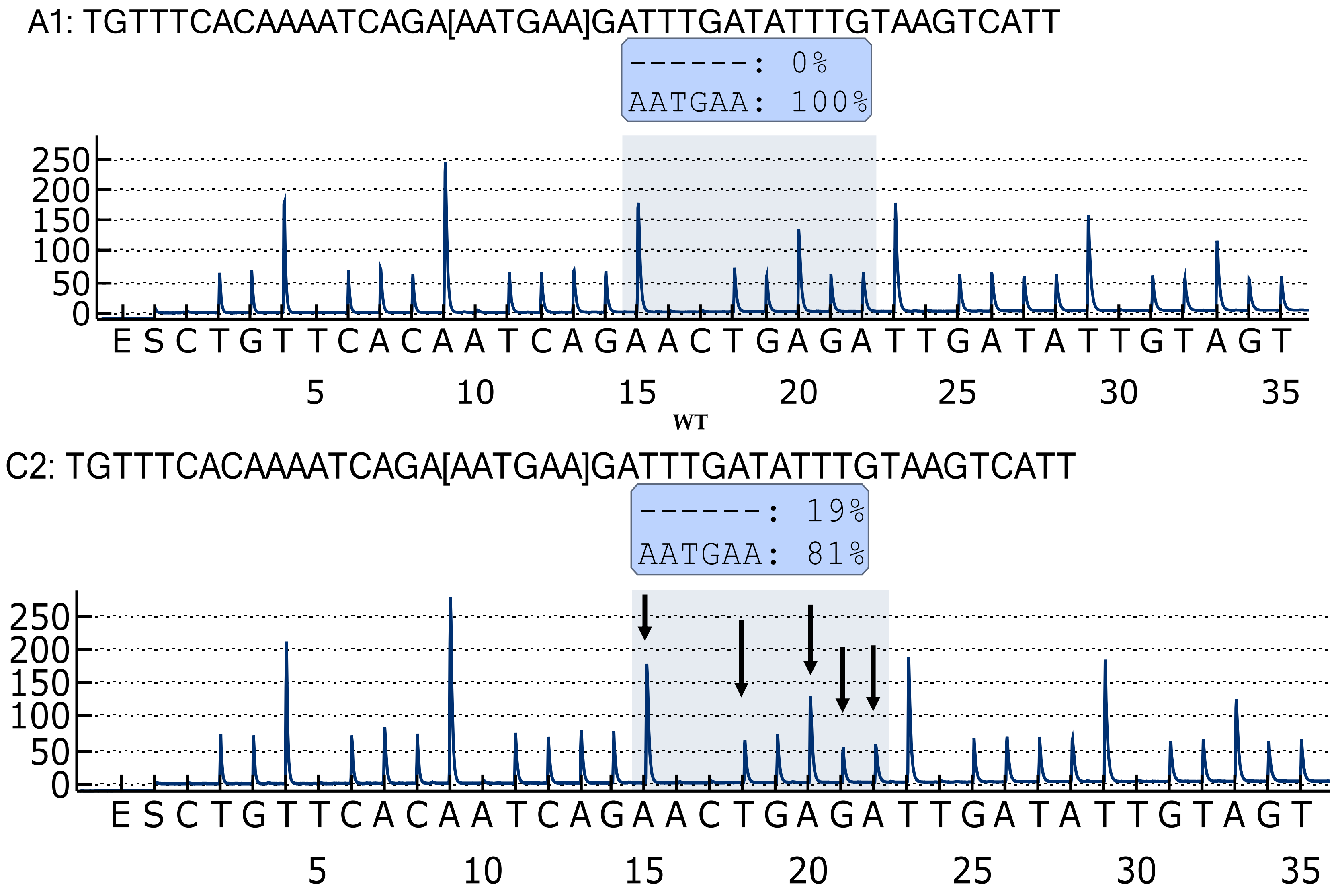

Mutant N542-E543del

(b)

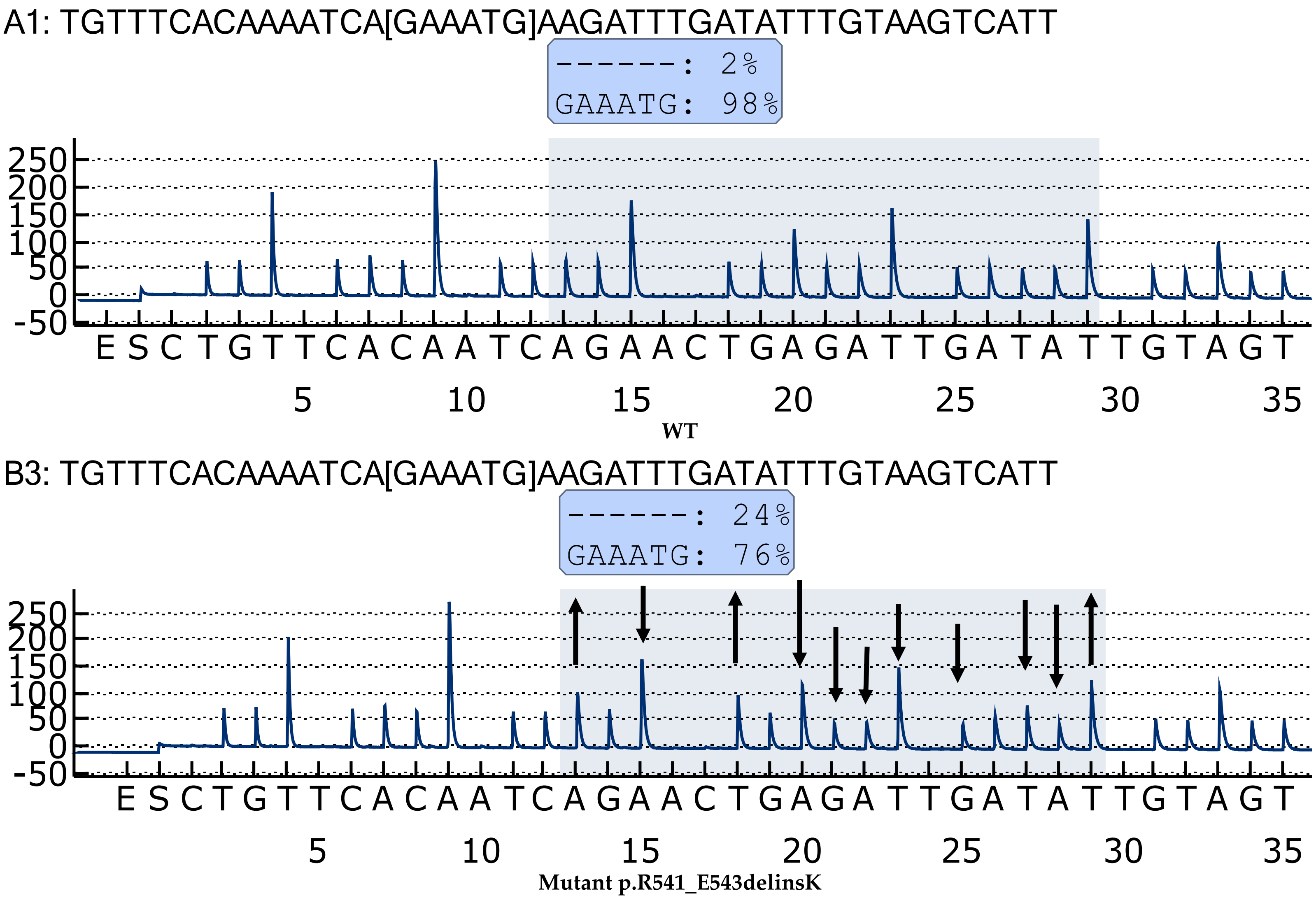
A1: TGTTTCACAAAATCA[GAAATG]AAGATTTGATATTTGTAAGTCATT
-------: 2%
GAAATG: 98%
250
200
150
100
50
0
-50
E S C T G T T C A C A A T C A G A A C T G A G A T T G A T A T T G T A G T
5
10
15
20
25
30
35
WT
B3: TGTTTCACAAAATCA[GAAATG]AAGATTTGATATTTGTAAGTCATT
-------: 24%
GAAATG: 76%
250
200
150
100
50
0
-50
E S C T G T T C A C A A T C A G A A C T G A G A T T G A T A T T G T A G T
5
10
15
20
25
30
35
Mutant p.R541_E543delinsK

(c)

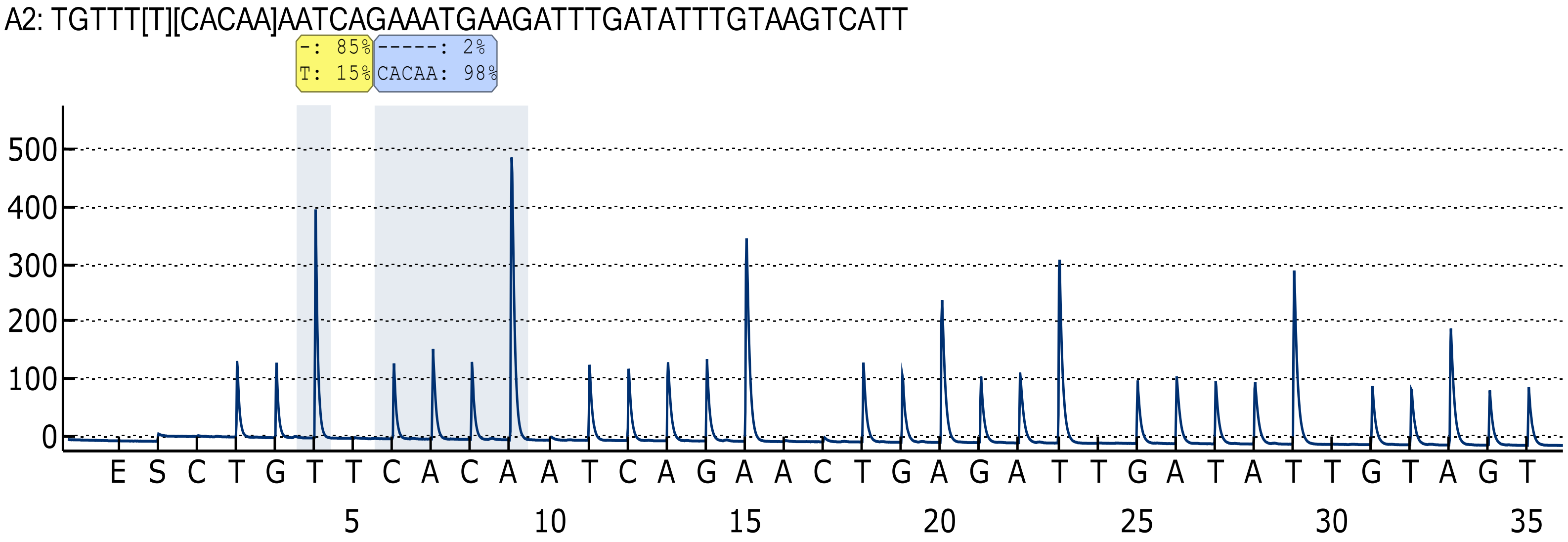

WT

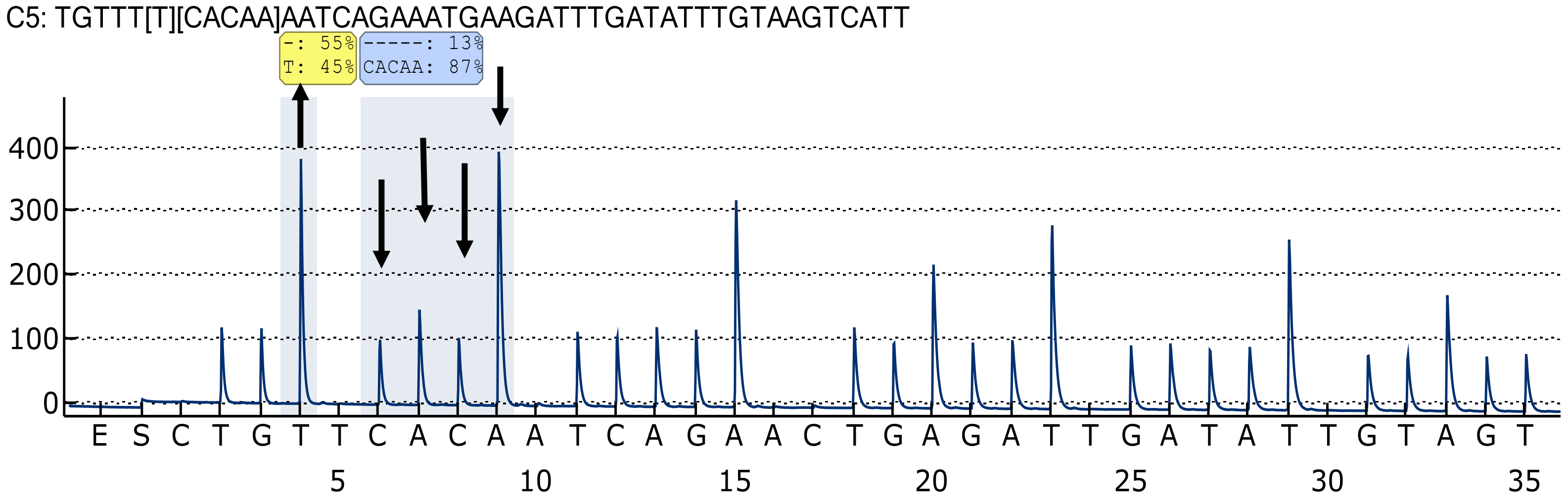

Mutant p.H538Nfs*4

(d)

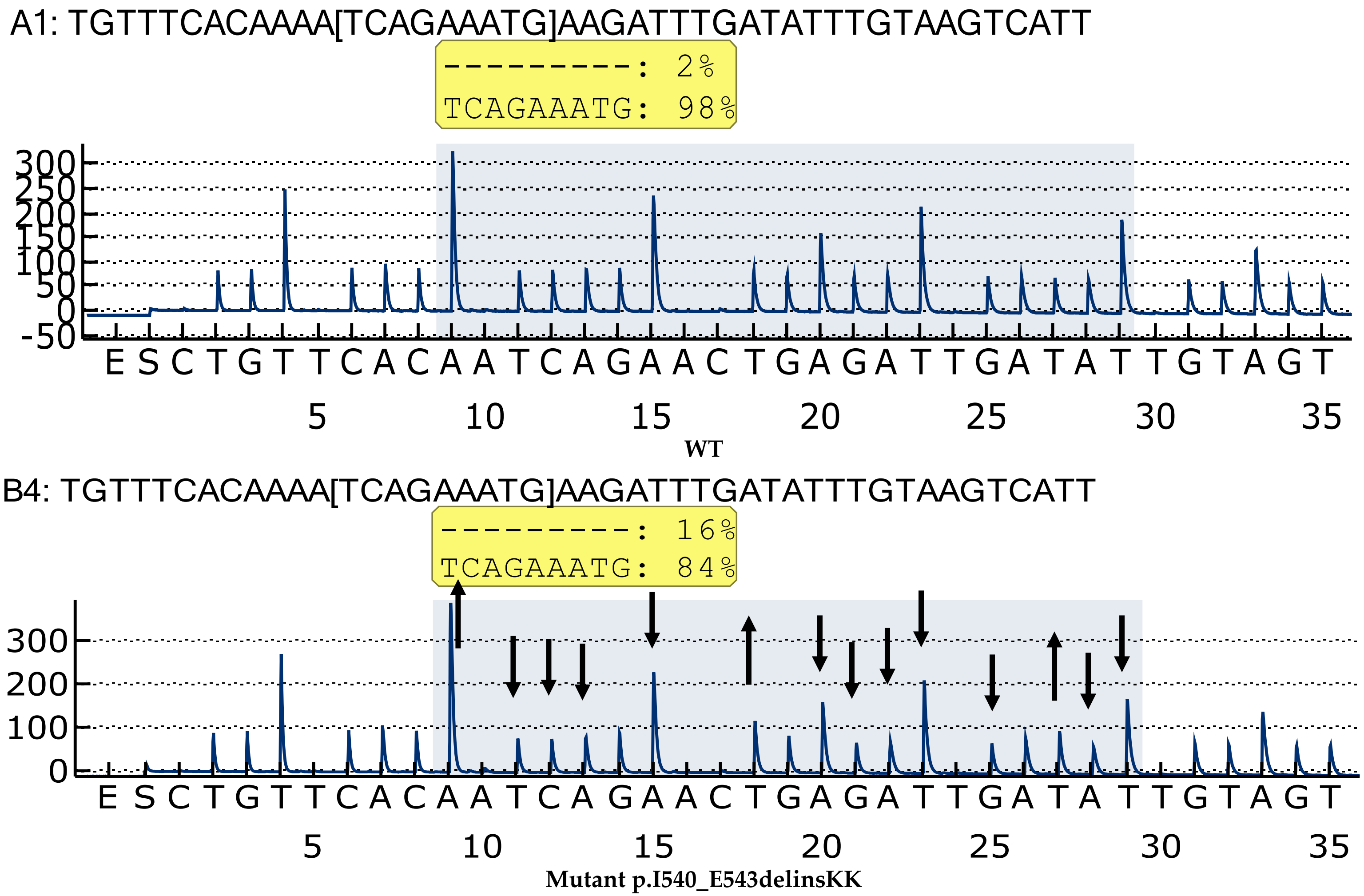

(e)

(a) - Sequence of p.F537_K539delinsL mutation, revealing TCACAA deletion, compared to sequence of wild type sample, allele burden mutation 35%; **(b)** - Sequence of p.N542-E543del mutation, revealing AATGAA deletion, compared to sequence of wild type sample, allele burden mutation 19%; **(c)** - Sequence of p.R541_E543delinsK mutation, revealing GAAATG deletion, compared to sequence of wild type sample, allele burden mutation 24; **(d)** - Sequence of p.H538Nfs*4 mutation, revealing CACAA deletion and TT insertion, compared to

sequence of wild type sample, allele burden mutation 13%; the TTTT homopolymer is marked in yellow («check») because there are difficulties in analyzing homopolymers using pyrosequencing, the software is unable to calculate the exact number of embedded nucleotides, and this result needs to be verified with care. The exact level of the mutant allele is recommended to be determined at position [CACAA]; **(e)** - Sequence of p.I540_E543delinsKK mutation, revealing TCAGAAATG deletion and AAA insertion, compared to sequence of wild type sample, allele burden mutation 13%; [TCAGAAATG] position is marked in yellow («check») because the uncertainty arises from the excessively long variable nucleotide sequence and the inability to analyze the homopolymer AAAAAA. The X-axis represents the sequence of nucleotides supplied into the reaction mixture, and the Y-axis represents signal level detected by the device. The peak height is proportional to the number of identical nucleotides consecutively incorporated. The nucleotide sequences used for mutation analysis are shown above pyrograms. The arrows indicate signals for nucleotides with the values changing in case of mutation. PyroMark Q24 v2.0.8 software performs quantitative analysis of variable nucleotide region automatically.
